# Reply to Apanovich and Weeks: Exceptions exist, as recognized in the paper, but are too rare to challenge the original findings

**DOI:** 10.1073/pnas.2509168122

**Published:** 2025-05-30

**Authors:** Gidon Eshel, Avi I. Flamholz, Alon Shepon, Ron Milo

**Affiliations:** ^a^Department of Environmental Science, Bard College, Annandale-on-Hudson, NY; ^b^Laboratory of Environmental Microbiology, The Rockefeller University, New York, NY 10065; ^c^The Steinhardt Museum of Natural History, Tel Aviv University, Tel Aviv 61390, Israel; ^d^Department of Plant and Environmental Science, Weizmann Institute of Science, Rehovot 7610001, Israel

We appreciate the comments (1).

The used sequestration statistics (2) are based on all meta-analysis data except from overstocked or overgrazed plots, because overgrazing is known to undermines soil carbon sequestration, placing grass-fed beef at a disadvantage. We excluded no data because they represent cropland. In retrospect, we now recognize that “exclusion”—by which we meant that we did not manually add individual grazed cropland estimates not included in the cited meta-analyses—was poor and confusing wording. Using the meta-analyses directly permits our estimates of the effect of grazing on soil carbon sequestration to “inherit” their rigor, endowing their condensed summaries—and thus Fig. 2*B*—with statistical representativeness.

In exceptional individual sites, grazing *may* indeed substantially enhance carbon sequestration, which we acknowledge. For example, the first Discussion sentence—“with realistic rangeland sequestration rates, the carbon intensity of *most* grass fed beef operations is in *most* cases higher than that of industrial beef”—emphasizes that the empirical findings are not universal, but potentially variable in some specific locations. However, the quantitative impact of these exceptional cases on the overall sequestration statistics is too small to meaningfully impact the paper’s title and detailed results.

While it is true that not all carbon-rich grasslands are readily convertible to cropland (2), the overwhelming majority are. This includes the grasslands on which high sequestration rates have been observed (reference 31 of the main paper and references 26, 31, and 32 of the *SI Appendix*). Only rare montane grasslands are remote and rugged enough to preclude cultivation, and their aggregated beef production is negligible. The “sod busting” history of the US Great Plains and similar Asian or South American grasslands, in which vast grassland biomes were plowed into grain-producing croplands, makes clear that nearly all expansive grasslands can be cultivated.

Similarly, while rotational or regenerative grazing *may sometimes* promote carbon sequestration, the three cited meta-analyses show (*SI Appendix*, Fig. S6) that this potential is infrequently realized. Instead, these meta-analyses show that grazing effects on soil carbon sequestration are so inconsistent and variable that they cannot be statistically distinguished from no effect (white squares in *SI Appendix*, Fig. S6).

The notion that because some grasslands are “already dedicated pasture[s],” they do not displace food production is false. Rather, we collectively pay an “opportunity cost” by electing not to use such grasslands to grow food for direct human consumption. What matters is not how those grasslands are used now, but the full scope of their potential uses, which includes rewilding and cultivation or some other societal uses such as solar panel installations.

Apanovich et al. (1) suggest that our work supports the notion that grazing “fertile lands” could nearly neutralize beef’s emissions. To the contrary, and as Pelletier et al. (reference 31 in the paper) makes clear, the empirical evidence is that grass feeding merely reduces somewhat the heavy carbon burden that beef production exerts.
